# Anti-citrullinated protein antibody specificities and pulmonary fibrosis in relation to genetic loci in early rheumatoid arthritis

**DOI:** 10.1093/rheumatology/keac280

**Published:** 2022-05-09

**Authors:** Mikael Brink, Lotta Ljung, Monika Hansson, Johan Rönnelid, Rickard Holmdahl, Karl Skriner, Guy Serre, Lars Klareskog, Solbritt Rantapää-Dahlqvist

**Affiliations:** Department of Public Health and Clinical Medicine/Rheumatology, University Hospital, Umeå; Department of Public Health and Clinical Medicine/Rheumatology, University Hospital, Umeå; Rheumatology Unit, Department of Medicine, Karolinska Institute at Karolinska University Hospital, Stockholm; Department of Immunology, Genetics and Pathology, Uppsala University, Uppsala; Medical Inflammation Research, Department of Medical Biochemistry and Biophysics, Karolinska Institute, Solna, Stockholm, Sweden; Department of Rheumatology and Clinical Immunology, Charite Universitätsmedizin, Berlin, Germany; Institut Toulousain des Maladies Infectieuses et Inflammatoires, UMR 1291 Inserm, 5051 CNRS, Université de Toulouse, Toulouse, France; Rheumatology Unit, Department of Medicine, Karolinska Institute at Karolinska University Hospital, Stockholm; Department of Public Health and Clinical Medicine/Rheumatology, University Hospital, Umeå

**Keywords:** RA, pulmonary fibrosis, autoantibodies, ACPA specificities, genetic loci

## Abstract

**Objectives:**

Pulmonary manifestations in RA are common comorbidities, but the underlying mechanisms are largely unknown. The added value of a multiplex of ACPA and genetic risk markers was evaluated for the development of pulmonary fibrosis (PF) in an inception cohort.

**Methods:**

A total of 1184 patients with early RA were consecutively included and followed prospectively from the index date until death or 31 December 2016. The presence of 21 ACPA fine specificities was analysed using a custom-made microarray chip (Thermo Fisher Scientific, Uppsala, Sweden). Three SNPs, previously found related to PF were evaluated, rs2609255 (*FAM13A*), rs111521887 (*TOLLIP*) and rs35705950 (*MUC5B*). ACPA and genetic data were available for 841 RA patients, of whom 50 developed radiologically defined PF.

**Results:**

In unadjusted analyses, 11 ACPA specificities were associated with PF development. In multiple variable analyses, six ACPA specificities were associated with increased risk of PF: vimentin (Vim)60–75, fibrinogen (Fib)β62–78 (72), Fibα621–635, Bla26, collagen (C)II359–369 and F4-CIT-R (*P* < 0.01 to *P* < 0.05). The number of ACPA specificities was also related to PF development (*P* < 0.05 crude and adjusted models). In multiple variable models respectively adjusted for each of the SNPs, the number of ACPA specificities (*P* < 0.05 in all models), anti-Vim60–75 (*P* < 0.05, in all models), anti-Fibβ62–78 (72) (*P* < 0.001 to *P* < 0.05), anti-CII359–369 (*P* < 0.05 in all models) and anti-F4-CIT-R AQ4 (*P* < 0.01 to *P* < 0.05), anti-Fibα621–635 (*P* < 0.05 in one) and anti-Bla26 (*P* < 0.05 in two) were significantly associated with PF development.

**Conclusion:**

The development of PF in an inception cohort of RA patients was associated with both presence of certain ACPA and the number of ACPA specificities and risk genes.

Rheumatology key messages

- Development of pulmonary fibrosis in rheumatoid arthritis is associated with certain ACPA fine specificities.
- The number of positive ACPA specificities increased the risk for development of pulmonary fibrosis.
- Presence of the autoantibodies associated with pulmonary fibrosis was unrelated to the risk genes.

## Introduction

RA is a chronic systemic inflammatory disease primarily affecting the joints, but with a noticeable frequency of severe extra-articular manifestations, especially lung disease [1]. Interstitial lung disease (ILD) with fibrosis in RA shares several characteristics with idiopathic pulmonary fibrosis (IPF), with a progressive course and poor survival [2]. The lungs have been proposed as a site of disease initiation with increased amounts of citrullinated peptides in lung tissue of smokers [3]. Thus, smoking in the presence of HLA-DRB1-shared epitope alleles may lead to the development of ACPA-positive RA [4]. However, infections and inflammation also increase citrullination of proteins, and citrullinated proteins have been found in lung tissue besides that from RA patients, in patients with idiopathic ILD [5, 6]. A broader ACPA repertoire was presented in RA-ILD suggesting a role for ACPA in the pathogenesis of ILD [7].

A strong genetic basis for IPF has been demonstrated in studies of familial aggregations [8] and in genome-wide association studies (GWASs) of the general population [9, 10] and of RA patients [11]. In a recent published study we found three SNPs, rs35705950 (*MUC5B*), rs111521887 (*TOLLIP*) and rs2609255 (*FAM13A*), to be significantly associated with pulmonary fibrosis (PF) in RA patients [12]. No significant relationship was found between anti-CCP2 antibodies and PF.

Here, we report the presence of ACPA fine specificities in relation to the development of PF and the three previously identified PF risk SNPs, analysed in our inception cohort of patients with RA followed prospectively within the catchment area of northern Sweden.

## Methods

The subjects and collection of clinical patient data and genetic analyses, as well as autoantibody detection, have been described previously [12, 13]. Briefly, an inception cohort of patients diagnosed with early RA (eRA) (symptomatic <12 months before diagnosis) according to the American Rheumatism Association classification criteria [14] were consecutively included in the study at the time of diagnosis (index date) between 1 January 1996 and 31 December 2016 at the five rheumatology clinics in northern Sweden. Clinical data, e.g. the 28-joint count and disease activity score (DAS28), were registered systematically and recorded at index date and at 6, 12, 18 and 24 months after diagnosis. Pharmacological treatments defined as corticosteroids and conventional synthetic DMARDs (csDMARDS; methotrexate, sulfasalazine, chloroquine, leflunomide, azathioprine, ciclosporin, mycophenolate mofetil and injectable gold salts) and biologic DMARDs (bDMARDs; abatacept, adalimumab, anakinra, etanercept, infliximab, rituximab, tocilizumab) were registered at the index date and continuously for the first 24 months. Smoking habits were registered as smoking ever *vs* non-smoking and as current smoker. Examinations and evaluations of the radiographs of the lungs were performed as previously described [12]. Data on high resolution CT (HRCT) examinations were collected during a 20 year period limiting the evaluation of the pulmonary manifestations to PF including reticular pattern, honeycombing or traction bronchiectasis of variable degree and with ground-glass in a few cases [15]. Thus, no further diagnostic evaluation was performed to identify and separate manifestations of ILD. Of the original included patients (*n* = 1184) in 50 patients no information on radiologic pulmonary examinations could be retrieved; these patients were excluded, leaving 1134 patients for further analyses [12]. In the remaining eRA cohort, the patients had received a diagnosis of PF at inclusion or during follow-up, in 96% of the cases based on HRCT findings and in two on plain X-rays after a mean (s.d.) of 6.2 (4.7) years after onset of RA.

Of the included 1134 pulmonary evaluated eRA patients, plasma samples were analysed in 841 patients for IgG-specific ACPA fine specificities, using the delta value from analysis of both the citrullinated peptides and their arginine-containing counterpart using a custom-made microarray chip (Thermo Fisher Scientific, ImmunoDiagnostics, Uppsala, Sweden). Autoantibodies against the following peptides were analysed: α-enolase peptide 5–21 (CEP-1), collagen type II (CII359–369, F4-R-CIT, F4-CIT-CIT and F4-CIT-R, all in triple helical conformation [16, 17]), fibrinogen (Fib)α36–50, Fibα563–583, Fibα580–600, Fibα621–635, Fibβ36–52, Fibβ60–74, Fibβ62–78(72), Fibβ62–78(74), filaggrin (Fil307–324), vimentin (Vim)2–17, Vim60–75 and hnRNP-A3 peptides (Bla26, Pept-1, Pept-5, PeptZ1 and PeptZ2) (presented in Supplementary Table S1, available at *Rheumatology* online). The methodology of analysing the antibodies is semi-quantitative, and thus dichotomized values of the antibodies are presented based on cut-off values set at the 98th percentile of 477 healthy controls; for more details see [13, 18]. At index date the analyses of RF used routine laboratory methods and those of anti-CCP2 antibodies were according to the manufacturer (EuroDiagnostica, Malmö, Sweden). All autoantibody analyses were done in plasma samples collected at the index date and preserved at −80°C until analysed. The antibody frequencies in the cohort with pulmonary examinations corresponded to the frequencies in the originally ACPA analysed cohort [13] (Supplementary Table S2, available at *Rheumatology* online).

GWAS genotyping of DNA samples from cases and controls was performed using the Global Screening Assay (GSA; Illumina, San Diego, CA, USA) to analyse 571 151 genome-wide single-nucleotide polymorphisms (SNPs) at deCode Genetics (Reykjavík, Iceland). The three loci containing SNPs found to be significantly associated with PF development were used for further analyses: rs35705950 (*MUC5B*), rs111521887 (*TOLLIP*) and rs2609255 (*FAM13A*) [12]. HLA-shared epitope (HLA-SE) (0401/0404/0405/0408 or 0101) was identified from GWAS data using imputation from a European reference panel; for details see [12].

### Statistics

Statistical analysis was performed using SPSS software (v. 27.0, IBM Corp, Armonk, NY, USA) and R software version 4.1.2 (R Foundation for Statistical Computing, Vienna, Austria [19]). Descriptive data were summarized and presented as proportions with means and standard deviation. Frequencies were compared using the chi-square test, and for continuous data Student’s *t*-test was used. Associations between PF and possible predictors including genetic markers were analysed using logistic regression analysis and presented as the odds ratio (OR) with 95% confidence interval (CI). Frequency of ACPA positivity was calculated for each specificity and the number of ACPA specificities for each case. A crude *P*-value is presented for the simple model based on the same subsets of samples as the multiple variable models for which the adjusted *P*-value is presented. The significance level was set at *P* < 0.05. For missing data, imputations have been performed using linear/logistic regression based on age, sex, smoking, methotrexate use from index date (*n* = 33) and DAS28 at inclusion (*n* = 15). DAS28 was also summarized during the first 24 months to area under curve for DAS28 (DAS28-AUC24).

### Ethics

The study complies with the Declaration of Helsinki and the Regional Ethics Committees at Umeå University, Sweden approved this study (No. Dnr 2017-432-32M, 2019-02039) and the patients signed an informed consent to participate.

## Results

### Presence of ACPA fine specificities in eRA with pulmonary fibrosis

Of the 841 eRA patients included in our inception cohort and analysed for presence of ACPA fine specificities, 50 (5.9%) had PF at the time of diagnosis or developed PF during follow-up. Of the 21 analysed ACPA specificities, 11 antibodies were significantly associated with PF in unadjusted logistic regression analyses, namely anti-Fil307–324 (CCP1), anti-Vim60–75, anti-Vim2–17, anti-CEP1, anti-Fibβ62–78 (72), anti-Fibα621–635, anti-Bla26, anti-Pept5, anti-CII359-369, anti-F4-CIT-CIT and anti-F4-CIT-R antibodies, as well as the number of ACPA specificities, but not presence or level of anti-CCP2 (Table 1). In multiple variable analyses adjusted for sex, age at RA diagnosis, smoking ever status and presence of RF, six of the ACPA specificities remained significantly associated with increased risk of PF development, namely anti-Vim60–75 [OR 2.13 (95% CI 1.07, 4.50), *P* < 0.05], anti-Fibβ62–78 (72) [OR 2.43 (95% CI 1.20, 4.67), *P* < 0.01], anti-Fibα621–635 [OR 2.12 (95% CI 1.12, 3.85), *P* < 0.05], anti-Bla26 [OR1.92 (95%CI 1.05, 3.51), *P* < 0.05], anti-CII359–369 [OR 1.92 (95% CI 1.04, 3.56), *P* < 0.05] and anti-F4-CIT-R antibodies [OR 2.57 (95% CI 1.38, 4.79), *P* < 0.01] (data not shown). A higher number of ACPA specificities in an individual increased the risk for PF development, with the mentioned adjustments [OR 1.07 (95% CI 1.01, 1.13), *P* < 0.05]. The relationships between certain of the ACPA specificities and PF did not result from higher frequencies of these antibodies (see Supplementary Table S2, available at *Rheumatology* online).

**Table 1:** Demographic and clinical data of the included patients with early RA, with and without pulmonary fibrosis

Variable	PF(*n* = 50)	No PF(*n* = 791)	OR (95% CI)	*P*-value
Age at RA diagnosis, mean (s.d.), years	63.90 (10.35)	56.98 (13.85)	1.05 (1.02, 1.07)	**<0.001**
Female, *n* (%)	29 (58.0)	548 (69.1)	0.61 (0.34, 1.10)	0.098
Ever-smoker, *n* (%)	38 (77.6)	505 (64.7)	1.90 (0.95, 3.77)	0.068
Current smoker, *n* (%)	11 (22.4)	167 (21.4)	0.97 (0.47, 1.83)	0.93
Anti-CCP2 antibody^+^, *n* (%)	39 (78.0)	535 (67.5)	1.69 (0.85, 3.35)	0.135
Anti-CCP2 level, mean (s.d.)	412.81 (523.80)	316.52 (624.42)	1.00 (1.00, 1.001)	0.337
RF^+^, *n* (%)	43 (86.0)	580 (73.1)	2.25 (0.996, 5.077)	**0.051**
HLA-shared epitope, *n* (%)	37 (74.0)	583 (73.7)	1.04 (0.85, 1.27)	0.72
DAS28 on index date, mean (s.d.)	4.76 (1.49)	4.69 (1.43)	1.03 (0.85, 1.24)	0.78
DAS28-AUC until PF or 24 months, mean (s.d.)	77.33 (33.17)	83.23 (22.18)	0.989 (0.977, 1001)	**0.082**
DAS28-AUC_24_, mean (s.d.)	90.00 (20.05)	83.23 (22.18)	1.014 (1.001, 1.027)	**0.038**
Treatment at index date, *n* (%)				
Glucocorticoids	28 (56.0)	463 (59.8)	0.85 (0.48, 1.52)	0.587
Methotrexate	35 (70.0)	630 (82.0)	0.51 (0.27, 0.97)	**0.039**
SNP				
rs2609255 G, MAF	0.35	0.23	1.79 (1.17, 2.74)	**0.008**
rs111521887 G, MAF	0.32	0.21	1.78 (1.15, 2.75)	**0.009**
rs35705950 T, MAF	0.22	0.12	2.17 (1.25, 3.75)	**0.005**
Frequency of ACPA positivity (95%CI)				
Anti-Fil307–324 (CCP1)	72.0 (57.9, 82.8)	55.9 (52.3, 59.2)	2.03 (1.08, 3.82)	**0.03**
Anti-Vim60–75	74.0 (60.0, 84.4)	57.1 (53.4, 60.3)	2.14 (1.12, 4.08)	**0.02**
Anti-Vim2–17	34.0 (20.4, 47.6)	21.6 (18.7, 24.5)	1.87 (1.02, 3.43)	**0.04**
Anti-Fibβ62–78 (72)	28.0 (17.2, 42.1)	12.8 (10.6, 15.2)	2.66 (1.39, 5.10)	**0.002**
Anti-CEP1	70.0 (56.8, 83.2)	54.0 (50.5, 57.5)	1.99 (1.07, 3.79)	**0.03**
Anti-Fibα621–635	58.0 (43.9, 70.9)	38.9 (35.4, 42.2)	2.17 (1.21, 3.87)	**0.008**
Anti-Bla26	48.0 (34.5, 61.8)	32.4 (29.1, 35.6)	1.93 (1.09, 3.43)	**0.023**
Anti-Pept5	68.0 (54.6, 81.4)	52.7 (49.2, 56.2)	1.91 (1.04, 3.51)	**0.04**
Anti-CII359–369	46.0 (32.7, 59.9)	27.7 (24.6, 30.8)	2.23 (1.25, 3.96)	**0.006**
Anti-F4-CIT-CIT	55.1 (40.1, 67.3)	39.2 (35.7, 42.5)	1.90 (1.06, 3.40)	**0.028**
Anti-F4-CIT-R	46.9 (32.7, 59.9)	23.8 (20.9, 26.8)	2.83 (1.58, 5.08)	**<0.001**
Number of ACPA positive specificities, mean (s.d.)	10.8 (6.1)	8.2 (6.5)	1.06 (1.01, 1.12)	**0.01**

*P*-values shown in bold indicate statistical significance. CI: confidence interval; Collagen type II: CII359–369, F4-CIT-CIT, F4-CIT-R; fibrinogen (Fib): Fibα621–635, Fibβ62–78 (72); filaggrin (Fil307–324); vimentin (Vim)60–75; and mutated proteins (Bla26). DAS28-AUC_24_: Area under curve for accumulated data for DAS28 during 24 months; MAF: minor allele frequency; OR: odds ratio; PF: pulmonary fibrosis.

### Presence of ACPA fine specificities and SNPs in eRA with pulmonary fibrosis

In subsequent models, the ACPA specificities that remained significantly associated with PF after adjustments were further analysed respectively adjusted for the three PF risk SNPs (rs35705950, rs111521887 and rs2609255) (Table 2). These SNPs were, as we have shown, significantly associated with PF in this patient cohort (Table 1, and ref. [12]). In the multiple variable models (adjusted for sex, age, ever smoker status, methotrexate at index date and RF status) and including each separately of the three SNPs, antibodies against Vim60–75, Fibβ62–78 (72) and F4-CIT-R and the number of ACPA specificities remained significantly associated with PF development (Table 2; *P* < 0.001 to *P* < 0.05). However, with the adjustments the association between anti-Fibα621–635 and PF was attenuated (Table 2).

**Table 2:** Association between RA with and without PF and ACPA specificities analysed in seven different models

	**Multiple variable regression analyses** ^a^					
	+rs2609255		+rs111521887		+rs35705950	
Variable	OR (95% CI)	*P*-value	OR (95% CI)	*P*-value	OR (95% CI)	*P*-value
Model 1						
Anti-Vim60–75	2.26 (1.09, 5.07)	**0.036**	2.28 (1.10, 5.14)	**0.035**	2.44 (1.06, 6.25)	**0.046**
SNP	1.74 (1.09, 2.74)	**0.018**	1.69 (1.06, 2.564)	**0.024**	2.08 (1.12, 3.70)	**0.015**
Model 2						
Anti-Fibβ62–78 (72)	2.85 (1.40, 5.59)	**0.003**	2.72 (1.32, 5.33)	**0.005**	3.48 (1.58, 7.39)	**0.001**
SNP	1.74 (1.09, 2.75)	**0.019**	1.59 (1.01, 2.48)	**0.041**	2.25 (1.20, 4.07)	**0.008**
Model 3						
Anti-Fibα621–635	1.92 (1.01, 3.72)	**0.047**	1.90 (1.00, 3.68)	0.052	1.95 (0.95, 4.11)	0.071
SNP	1.71 (1.07, 2.69)	**0.023**	1.63 (1.03, 2.55)	**0.034**	2.01 (1.08, 3.57)	**0.021**
Model 4						
Anti-F4-CIT-R	2.46 (1.28, 4.74)	**0.007**	2.52 (1.31, 4.84)	**0.005**	2.34 (1.13, 4.85)	**0.021**
SNP	1.51 (0.93, 2.42)	0.087	1.53 (0.95, 2.41)	**0.074**	1.92 (1.02, 3.44)	**0.033**
Model 5						
Anti-Bla26	1.89 (1.00, 3.57)	**0.049**	1.90 (1.01, 3.59)	**0.046**	1.80 (0.89, 3.67)	**0.10**
SNP	1.72 (1.07, 2.72)	**0.023**	1.65 (1.04, 2.58)	**0.029**	2.02 (1.10, 3.60)	**0.019**
Model 6						
Anti-CII359–369	2.05 (1.07, 3.94)	**0.030**	2.25 (1.17, 4.32)	**0.014**	2.10 (1.02, 4.32)	**0.042**
SNP	1.66 (1.04, 2.63)	**0.031**	1.72 (1.08, 2.69)	**0.019**	2.06 (1.12, 3.65)	**0.016**
Model 7						
Number of ACPA specificities	1.07 (1.01, 1.13)	**0.025**	1.07 (1.01, 1.13)	**0.021**	1.07 (1.01, 1.15)	**0.025**
SNP	1.60 (0.99, 2.55)	0.050	1.60 (0.99, 2.53)	**0.047**	1.95 (1.04, 3.50)	**0.029**

^a^ Adjusted for; sex, age at onset, ever smoker status, methotrexate and RF status at index date. *P*-values shown in bold indicate statistical significance. CI: confidence interval; Collagen type II: F4-Cit-R; fibrinogen (Fib): Fibα621–635, Fibβ62–78 (72); vimentin (Vim)60–75. OR: odds ratio.

No association was found between either of the three SNPs and positivity for any of the ACPA fine specificities (data not shown).

## Discussion

This exploratory study of a consecutively included inception cohort of patients with eRA is the first study to analyse both PF-related genetic risk loci and ACPA fine specificities for the PF development in RA patients. Associations were found between certain ACPA specificities and PF risk, irrespective of the risk genotype. The number of ACPA specificities was also associated with PF development, independent of presence of the selected risk genotypes. However, we did not find a significant association between PF and anti-CCP2 positivity or levels, but the findings of ACPA fine specificities are in line with a previous publication where the number of high levels of ACPAs was associated with the degree of PF [7]. Higher levels of all ACPA specificities analysed by Giles *et. al.* [7] showed significant association with ILD, including specificities to fibrinogen and vimentin, as shown in the present work although not using exactly the corresponding peptides. Further, Giles *etal*. presented an association between the level of antibodies against α-enolase and ILD, while no association of this antibody could be found with PF in our cohort (*P* = 0.067, *P*-value in unadjusted model). Higher frequency or higher level, respectively, of anti-filaggrin antibodies [Fil307–324 (CCP1)] and of anti-enolase 1A cyclic and anti-CEP1 antibodies showed significant associations in each of the two studies with the outcome measure, PF and ILD, respectively. In our study the significant association was lost after the mentioned adjustments (for anti-filaggrin antibodies *P* < 0.030 and 0.065 and for anti-CEP1 0.031 and 0.057 in unadjusted and adjusted models, respectively). An expanded ACPA repertoire was suggested by Giles *et al.* as a distinct feature of antibody associations with ILD, e.g. expanded ACPA specificities were most strongly associated with features of fibrosis, while anti-CCP2 level indicated ILD outcome [7]. These results are in line with our findings as we have focused on PF separately and not the full pattern of ILD, and we found no association with anti-CCP2 antibodies and PF. The antibodies associated with PF in the present study were not among those of the highest frequency in the patient cohort. Three of the antibodies were directed against collagen type II, which is the main structural protein of hyaline cartilage. Another two were respectively directed against the α and β chain of fibrinogen. Presence of the antibodies associated with PF was unrelated to the risk genes and could represent a separate process ongoing in the lung parenchyma or just representing a para-phenomenon.

The strength of the current study is that the cohort of eRA patients was unselected and originated from a homogeneous population of northern Sweden. Almost all (95%) individuals diagnosed with early RA within the catchment area of northern Sweden were willing to participate in the study. Furthermore, X-rays of the lungs of the patients were routinely performed at inclusion, providing baseline information about the lungs.

We have, however, identified some limitations of this study. The HRCT examinations in this study were not performed randomly or on all included patients, but for those with abnormalities on the plain X-rays or with clinical indications of development of defined symptoms. Further, the HRCT examinations were performed over a period of almost 20 years, and hence methodological improvements during this time could affect the results. Consequently, we refrained from further diagnostic evaluations of ILD besides PF.

In this study we have shown that development of PF in patients with RA is associated to the number of ACPA specificities and to certain ACPA fine specificities independently of previously identified PF-related genetic risk loci.

## Supplementary Material

keac280_Supplementary_Data

## Data Availability

The data underlying this article cannot be shared publicly due to the privacy of individuals that participated in the study. The data will be shared on reasonable request to the corresponding author.
