# Supplementary material for: Anti-citrullinated protein antibody specificities and pulmonary fibrosis in relation to genetic loci in early rheumatoid arthritis
Source: Rheumatology (Oxford). 2022 May 9;61(12):4985–90. doi: 10.1093/rheumatology/keac280 (PMC9729003; doi:10.1093/rheumatology/keac280)
Supplement: keac280_Supplementary_Data [file keac280_supplementary_data.docx]

**Supplementary Table S1. Peptide sequence and frequency of the 21 analysed ACPA fine specificities, in 841 early RA patients with and without pulmonary fibrosis (PF).**

|  | Citrullinated Peptide | Amino acids | Amino acid sequence^†^ | PF  Frequency  (CI 95%) | No PF  Frequency  (CI 95%) |
| --- | --- | --- | --- | --- | --- |
| α-Enolase | Eno5-21 (CEP-1) | 5-21 | CKIHA(cit)EIFDS(cit)GNPTVEC (cyclic)1 | 70.0 (55.9, 81.1)* | 54.0 (50.5, 57.4) |
| Collagen II | C1-CIT-CIT | 359-369 | (GPO)5-GA(cit)GLTG(cit)PGDA(GPO)2-GKKYG | 46.0 (32.7, 59.9)** | 27.7 (24.7, 30.9) |
|  | F4-R-CIT | 933 | (GPO)5-GDKGEAGEOGERGLKGH(cit)GFTGLQ(GPO)2 | 54.0 (40.1, 67.3)* | 48.3 (44.8, 51.8) |
|  | F4-CIT-CIT | 927, 933 | (GPO)5-GDKGEAGEOGE(cit)GLKGH(cit)GFTGLQ(GPO)2 | 54.0 (40.1, 67.3)** | 39.2 (35.8, 42.6) |
|  | F4-CIT-R | 927 | (GPO)5-GDKGEAGEOGE(cit)GLKGHRGFTGLQ(GPO)2 | 46.0 (32.7, 59.9)*** | 23.8 (20.9, 26.9) |
| Fibrinogen | Fibα36-50 | 36-50 | GP(cit)VVE(cit)HQSACKDS | 36.0 (23.9, 50.2) | 26.0 (23.1, 29.2) |
|  | Fibα563-583 | 563-583 | HHPGIAEFPS(cit)GKSSSYSKQF | 46.0 (32.7, 59.9) | 37.8 (34.5, 41.2) |
|  | Fibα580-600 | 580-600 | SKQFTSSTSYN(cit)GDSTFESKS | 30.0 (18.9, 44.1) | 20.4 (17.7, 23.3) |
|  | Fibα621-635 | 621-635 | (cit)GHAKS(cit)PV(cit)GIHTS | 58.0 (43.9, 70.9)** | 38.9 (35.6, 42.4) |
|  | Fibβ36-52 | 36-52 | NEEGFFSA(cit)GHRPLDKK | 70.0 (55.9, 81.1) | 56.0 (52.5, 59.4) |
|  | Fibβ62-78 (72) | 62-78 | APPPISGGGY(cit)ARPAKAAAT | 28.0 (17.2, 42.1)** | 12.8 (10.6, 15.3) |
|  | Fibβ62-78 (74) | 62-78 | APPPISGGGYRA(cit)PAKAAAT | 36.0 (23.9, 50.2) | 25.4 (22.5, 28.6) |
|  | Fibβ60-74 | 60-74 | (cit)PAPPPISGGGY(cit)A(cit) | 70.0 (55.9, 81.1) | 63.6 (60.2, 66.9) |
| Filaggrin | Fil307-324 (CCP1) | 307-324 | SHQEST(cit)GRSRGRSGRSGS (cyclic) | 72.0 (57.9, 82.8)* | 55.9 (52.4, 59.3) |
| Vimentin | Vim2-17 | 2-17 | ST(cit)SVSSSSY(cit)(cit)MFGG | 34.0 (22.2, 48.2)* | 21.6 (18.9, 24.6) |
|  | Vim60-75 | 60-75 | VYAT(cit)SSAV(cit)L(cit)SSVP | 74.0 (60.0, 84.4)* | 57.1 (53.7, 60.6) |
| hnRNP | Bla-26 | (proprietary) | (proprietary) | 48.0 (34.5, 61.8)* | 32.4 (29.2, 35.7) |
|  | PeptZ1 | (proprietary) | (proprietary) | 56.0 (42.0, 69.1) | 46.3 (42.8, 49.8) |
|  | PeptZ2 | (proprietary) | (proprietary) | 60.0 (45.8, 72.7) | 50.2 (46.7, 53.7) |
|  | Pept-1 | (proprietary) | (proprietary) | 30.0 (18.9, 44.1) | 24.7 (21.8, 27.8) |
|  | Pept-5 | (proprietary) | (proprietary) | 68.0 (53.8, 79.5)* | 52.7 (49.2, 56.2) |
| ^†^For further references for each antibody see Boman et. al. ref. 10.  ^‡^P-value using pearson chi-squared, *** P< 0.001, ** P<0.01, * P<0.05. | | | | | |

**Supplementary Table S2.** Frequency of citrullinated/mutated antibodies in 1011 of the early RA patients at index date, and for 841 of the patients who were pulmonary evaluated presented with decreasing frequencies. In bold the ACPA reactivities associated with pulmonary fibrosis. The frequencies are presented with decreasing values.

| Antibody against citrullinated/mutated  peptides | All eRA patients^1^  N=1011  n (%) | All eRA patients evaluated for pulmonary  involvements, n=841  n (%) |
| --- | --- | --- |
| Anti-CCP2 | 680 (67.5) | 575 (68.4) |
| Fibβ60-74 | 645 (63.1) | 538 (64.0) |
| **Vim60-75** | 578 (56.6) | 489 (58.1) |
| Fibβ36-52 | 562 (55.9) | 478 (56.8) |
| **Fil307-324 (CCP1)** | 561 (54.9) | 478 (56.8) |
| αEno5-21 (CEP-1) | 551 (53.9) | 462 (54.9) |
| Pept-5 | 530 (51.9) | 451 (53.6) |
| PeptZ2 | 506 (49.5) | 427 (50.8) |
| F4-R-CIT | 492 (48.1) | 409 (48.7) |
| PeptZ1 | 480 (47.0) | 394 (46.8) |
| **F4-CIT-CIT** | 411 (40.2) | 337 (40.2) |
| **Fibα621-635** | 398 (38.9) | 337 (40.1) |
| Fibα563-583 | 387 (37.9) | 322 (38.3) |
| **Bla-26** | 338 (33.1) | 280 (33.3) |
| **CII359-369** | 306 (29.9) | 242 (28.8) |
| Fibα36-50 | 261 (25.8) | 224 (26.6) |
| **F4-CIT-R** | 264 (25.8) | 211 (25.1) |
| Fibβ62-78 (74) | 262 (25.6) | 219 (26.0) |
| Pept-1 | 250 (24.5) | 210 (25.0) |
| Vim2-17 | 222 (21.7) | 188 (22.4) |
| Fibα580-600 | 205 (20.1) | 176 (20.9) |
| **Fibβ62-78 (72)** | 136 (13.3) | 115 (13.7) |

^1^presented in ref.13.

The antibodies sorted α-enolase peptide 5-21 (CEP-1), Collagen type II (CII359-369, F4-R-CIT, F4-CIT-CIT, F4-CIT-R), Fibrinogen (Fib) α36-50, Fibα5673-583, Fibα580-600, Fibα621-635, Fibβ36-52,

Fibβ62-78 (72), Fibβ62-78 (74), Fibβ60-74, Filaggrin (Fil307-324), Vimentin (Vim) 2-17, Vim60-75

and mutated proteins (Bla26, Pept-1, Pept-5, PeptZ1, PeptZ2).
